# Intravenous administration of Penicillin results in therapeutic intravitreal levels in chronic postoperative endophthalmitis

**DOI:** 10.1186/s12348-020-00232-0

**Published:** 2021-01-22

**Authors:** Chloe Thabet, Chloe C. C. Gottlieb, Bernard R. Hurley, Guijun Zhang, Adeel Sherazi, Jonathan B. Angel

**Affiliations:** 1 Faculty of Medicine, University of Ottawa, Ottawa, ON Canada; 2 Department of Ophthalmology, University of Ottawa, Ottawa, ON Canada; 3 Ottawa Hospital Research Institute, Ottawa, ON Canada; 4 Department of Medicine, Division on Infectious Diseases, University of Ottawa, Ottawa, ON Canada

## Abstract

**Importance:**

The role of systemic antibiotics in the treatment of bacterial endophthalmitis remains controversial. While penicillin is a highly effective antibiotic against bacteria that frequently cause endophthalmitis, the ability of systemically administered Penicillin G to penetrate into the vitreous at adequate therapeutic concentrations has not been studied. Its role in the treatment of endophthalmitis, particularly for bacteria for which it is the antibiotic of choice, therefore remains unknown.

**Objective:**

We sought to determine whether intravenous administration of Penicillin G leads to adequate therapeutic concentrations in the vitreous for the treatment of bacterial endophthalmitis.

**Design and setting:**

This study was conducted in an ambulatory setting, at the Ottawa Hospital Eye Institute, a university-affiliated tertiary care center, where a 77-year old gentleman with chronic post-cataract surgery *Actinomyces neuii* endophathalmitis was treated with intravenous Penicillin G (4 × 10^6^ units every 4 h) and intravitreal ampicillin (5000μg/0.1 m1).

**Main outcomes and measures:**

Intravitreal concentration of Penicillin G and ampicillin were obtained at the time of intraocular lens removal, measured by high-performance liquid chromatography.

**Results:**

The intravitreal concentration of penicillin and ampicillin was 3.5μg/ml and 0.3μg/ml, respectively. Both the concentration of penicillin and ampicillin were within the level of detection of their respective assays (penicillin 0.06-5μg/ml, ampicillin 0.12–2.5μg/ml).

**Conclusion and relevance:**

This study shows that intravenous Penicillin G administered every four-hours allows for adequate intravitreal concentrations of penicillin. Future studies are required to determine if the results of this study translate into improved clinical outcomes.

## Introduction

Postoperative bacterial endophthalmitis is an uncommon, but very serious, complication of intraocular surgery [1]. Empiric antibiotic regimens commonly include intravitreal injections of vancomycin and ceftazidime for broad-spectrum coverage of Gram-positive and Gram-negative organisms [1]; however, no well supported treatment guidelines exist for the management of non-endogenous bacterial endophthalmitis [2]. The role of systemic antibiotics remains controversial [2].

The foundation of post-cataract surgery endophthalmitis management is based on the Endophthalmitis Vitrectomy Study (EVS), conducted over 25 years ago and which aimed to determine the role of intravenous antibiotics in the management of acute post-operative bacterial endophthalmitis [3]. In this study, the addition of intravenous to intravitreal antibiotics provided no benefit in visual acuity or media clarity [3]. Generalizability of these results, however, poses several challenges. First, patients with chronic or severe endophthalmitis were excluded from study. Second, the systemic antibiotics used (ceftazidime and amikacin) have poor activity against common Gram-positive organisms seen in endophthalmitis, with poor penetration into the vitreous (specifically amikacin). Third, the duration of systemic antibiotics (5–10 days), might have been too short to confer any benefit [2]. Since publication of the EVS, there has been little new literature to guide systemic antibiotics for the management of endophthalmitis [2].

Penetration of systemically administered antimicrobials into the intraocular space, through the blood-retinal barrier (BRB) is limited by the retinal pigment epithelium (RPE) and the tight junctions of retinal capillaries. Diffusion of antibiotics into the vitreous is restricted by poor vascularization and a small diffusion surface. Consequently, lower drug concentrations occur in the vitreous compared to the plasma [4]. Bypassing the BRB via intravitreal administration has traditionally been preferred and is the mainstay of treatment for bacterial endophthalmitis. Data derived from studies in humans suggest that carbapenems, linezolid, daptomycin, and high-dose moxifloxacin achieve adequate intraocular levels when administered intravenously [2]. Studies in rabbits also suggest that systemic administration of imipenem, ciprofloxacin, cefazolin, ceftazidime and vancomycin achieve adequate levels in the eye but extrapolation of this information to humans must be done with caution [5].

Penicillin is a highly effective antimicrobial against the predominant organisms seen in bacterial endophthalmitis and is the drug of choice for most streptococci and many anaerobic bacteria. However, the intravitreal penetration of systemically administered penicillin has not been studied. Based on similar properties between the blood-brain-barrier and the BRB, it is presumed that penetration of intravenous penicillin into the vitreous space is minimal [2]. To our knowledge, we are the first to report intravitreal levels of penicillin following systemic administration of Penicillin G in a patient with chronic postoperative *Actinomyces neuii* endophthalmitis.

## Methods

Analyses of intravitreal levels of penicillin and ampicillin in a patient with chronic post-cataract surgery endophthalmitis caused by *Actinomyces neuii* and requiring vitrectomy with lens removal were performed*.* A total of two intravitreal doses of ampicillin were given one-week apart. At the time of lens removal, one-week into treatment with intravenous Penicillin G (4 × 10^6^ units every 4 h) and 48 h post last dose of intraocular ampicillin (5000μg/0.1 ml), a sample of vitreous fluid was collected and stored at − 80^0^ C until the time of analysis. Penicillin and Ampicillin levels were measured by a validated liquid chromatography with tandem mass spectrometry method. The vitreous sample was pretreated with Acetonitrile for protein precipitation, and penicillin, ampicillin and internal standard (6,7-Dimethyl-2,3-di (2-pyridyl)-quinoxaline) were separated by high performance liquid chromatography with a C18 column. They were quantified by a triple quadruple mass spectrometer. Penicillin was linear between 0.06-5μg/ml and ampicillin was linear between 0.12–2.5μg/ml.

## Results

The intravitreal concentration of penicillin following 1 week of intravenous therapy, with associated clinical improvement, was 3.5μg/ml (Table 1). With every four-hour dosing, this is at a time by which stable intraocular levels can be expected to have been achieved. The intravitreal concentration of ampicillin 48 h after the last administered intravitreal dose was 0.3μg/ml, marginally above the level of detection of the assay (Table 1).

**Table 1:** Intravitreal Penicillin and Ampicillin Concentrations

Drug	Route	Dosing regimen	Intravitreal concentration	Range of quantitation
Penicillin	Intravenous	4 million U q4h	3.5μg/ml^a^	0.06-5μg/ml
Ampicillin	Intravitreal	5 mg (5000μg/0.1 ml) last administered 48 h prior	0.3μg/ml^a^	0.12–2.5μg/ml

^a^Levels were obtained from the same sample at the time of lens removal

## Discussion

Penicillin has a broad spectrum of activity against Gram-positive organisms and provides excellent activity against Streptococci and organisms commonly involved in bacterial endophthalmitis. However, data are lacking regarding the distribution of intravenous penicillin into the vitreous.

In two studies conducted in 1966 and 1967, the vitreal concentration of semi-synthetic penicillin derivatives oxacillin and methicillin were studied following systemic administration. In both cases, vitreal concentrations were below known therapeutic concentrations [6, 7]. A rabbit study evaluated the total concentration of penicillin in ocular tissues and found extremely high levels of penicillin in all ocular tissues except the lens and vitreous [8]. Similarly, inadequate levels of ampicillin were found in the vitreous of rabbit eyes following administration of intravenous ampicillin of 50 mg/kg [9]. In contrast, in 1998, Robinet et al. demonstrated the ability of piperacillin to adequately penetrate the vitreous cavity in humans, at concentrations sufficient to kill Gram-positive organisms in inflamed eyes only [10].

Adequate therapeutic concentrations of antimicrobials are determined by the minimum inhibitory concentration (MIC) of an antimicrobial against a specific pathogen. In the case of postoperative endophthalmitis, *S. pneumoniae* and *S. viridans* are amongst the most common causative streptococci [11]. Typical MICs of penicillin against *S. pneumoniae* and *S. viridans* range from 0.25-1μg/ml [12] and 0.125-4μg/ml [13] respectively. Most importantly, the MIC of penicillin against most strains of Actinomyces ranges from 0.063-2μg/ml [14]. As every four-hour dosing of intravenous penicillin would be expected to achieve constant levels of intravitreal penicillin, it reasonable to conclude that concentrations of penicillin achieved in the vitreous are sufficient for the treatment of common causes of post-operative endophthalmitis, including that caused by *Actinomyces*.

This study is the first to report adequate vitreal levels of penicillin against common endophthalmitis pathogens following intravenous administration. In this case, intravenously administered penicillin likely provided elevated intravitreal concentrations as a result of constant and continued diffusion from plasma. Anionic drugs including beta-lactams are subject to accelerated clearance via active transport across the BRB which, in this case, may have further promoted diffusion from the plasma into the vitreous cavity following the concentration gradient [15]. Our study found elevated levels of penicillin in the intravitreal space of an inflamed eye following intravenous administration, as seen with piperacillin [10]. This correlates well with studies that have found increased penetration of systemic antibiotics in inflamed eyes versus their non-inflamed counterparts [10, 15]. In inflamed eyes, drugs cleared by the posterior route, such as beta-lactams, demonstrate delayed clearance due to compromise of the RPE pump [15]. An extended half-life may have contributed to the elevated concentration of vitreal penicillin seen in our patient.

## Conclusion

To successfully treat endophthalmitis, rapid administration of antibiotics to the posterior segment is necessary to prevent vision loss. In this report, we have shown that intravenous penicillin administered every 4 h allows for adequate concentrations in the vitreous. Future studies are required to determine if these favourable pharmacokinetics translate into improved clinical outcomes along with the potential benefit of limiting intraocular injections.

## Data Availability

All available results and data is provided in this report.

## Abbreviations

EVS: Endophthalmitis Vitrectomy Study
BRB: Blood-retinal barrier
RPE: Retinal pigment epithelium
MIC: Minimum inhibitory concentration
